# Both Chloroquine and Lopinavir/Ritonavir Are Ineffective for COVID-19 Treatment and Combined Worsen the Pathology: A Single-Center Experience with Severely Ill Patients

**DOI:** 10.1155/2021/8821318

**Published:** 2021-02-06

**Authors:** Fernando Sevilla-Castillo, Oscar J. Roque-Reyes, Fernanda Romero-Lechuga, Mario F. Gómez-Núñez, Mariel Castillo-López, Diana Medina-Santos, Perla Oriana Román, Jorge Rafael Flores-Hernández, Juan Daniel Méndez-Coca, Daniela Montaño-Olmos, Karla Cecilia Farfán-Lazos, Miranda Tobón-Cubillos, América Viveros-Hernández, Leonardo Torres-Ortega, Karla Y. Hernández-Skewes, Guillermo Montiel-Bravo, Shannat Ortega-Rodríguez, Alberto N. Peón

**Affiliations:** ^1^Laboratorio Santiago Ramón y Cajal, Sociedad Española de Beneficencia, Pachuca, Hidalgo, Mexico; ^2^Área Académica de Medicina, Universidad Autónoma del Estado de Hidalgo, Mexico; ^3^Facultad de Medicina, Universidad Nacional Autónoma de México, Mexico; ^4^Facultad de Medicina, Benemérita Universidad Autónoma de Puebla, Mexico; ^5^Universidad Anáhuac, Puebla, Mexico; ^6^Escuela Superior de Apan, Universidad Autónoma del Estado de Hidalgo, Mexico

## Abstract

The off-label use of antiviral and antimalarial drugs has been considered by many researchers as a fast and relatively safe alternative to provide therapeutic options to treat COVID-19, but the assessment of such drug-specific effectiveness in this regard is far from complete. Especially, the current body of knowledge about COVID-19 therapeutics needs more data regarding drug effectiveness and safety in the severely ill patients with comorbidities. In the present article, we retrospectively analyze data from 61 patients that received treatment with chloroquine, lopinavir/ritonavir, both drugs administered together, or a standard treatment with no antiviral drugs, and the study was carried in severely ill patients. We found that either drug is ineffective at treating COVID-19, as they are not able to reduce hospitalization length, mortality, C-reactive protein (CRP), lactate dehydrogenase (LDH), d-Dimer, or ferritin, or to enhance gasometric parameters, lymphocytes, total leukocytes, and neutrophil levels, whereas both drugs administered together decrease circulating lymphocytes, increase LDH and ferritin levels, and more importantly, enhance mortality. In this way, our results show that both drugs are ineffective and even potentially harmful alternatives against SARS-CoV-2.

## 1. Introduction

Severe acute respiratory syndrome coronavirus 2 (SARS-CoV-2), which is the causative agent of the coronavirus disease (COVID-19), has caused more than 51 million infections and more than one million deaths worldwide in less than a year [1], also causing a severe economic impact in every affected nation [2].

The current pharmacopeia lacks a specific treatment for such an emerging disease, but the rapid spread of the virus has produced an urgency to develop novel pharmacological therapies, either by repurposing widely available drugs or by developing novel-specific therapeutics. However, the emergency of the situation calls for drugs that may be readily available and have a known safety profile, so that the former focus has gathered most of the attention [3], and despite the fact that COVID-19 is a complex disease that requires antiplatelet and anti-inflammatory treatments to be administered along with antiviral agents, the latter treatments are gathering most of the attention because these drugs attack the etiological factor of the disease.

Lopinavir alone [4] or on combination with ritonavir (L/R) [5], and especially chloroquine (Chlo) or hydroxycholoquine with or without azithromycin combinations [6, 7], have been among the most studied drugs. Lopinavir is a peptidomimetic molecule that inhibits the enzyme viral 3-chymotrypsin-like protease (3CL^pro^) by the formation of a hydroxyethylene scaffold that mimics the peptide linkage typically targeted by such viral protease, but cannot be cleaved, and thus binds to the active site of the enzyme [8]. This drug combination has been essential to treat human immunodeficiency virus (HIV) infections, and like HIV, SARS-CoV-2 expresses the 3CL^pro^ enzyme to control its replication [9], thus providing a rationale for the use of such drug in COVID-19. Moreover, the fact that the L/R has been shown to inhibit this coronavirus enzyme with enhanced affinity [10] pinpoints at a possible role for its use in the treatment of COVID-19.

On the other hand, chloroquine has been shown to deter the *in vitro* virus-host cell fusion by interfering with the glycosylation of the ACE2 protein [11–13]. Moreover, after penetration, the virus releases its genome and some enzymes into the cytoplasm by fusing with the lysosomal membrane, in a process aided by the acidic pH of the aforementioned cell structure. In concordance, chloroquine has been shown to inhibit this process in other enveloped virus-dependent infections, like Chikungunya and Dengue, by the means of lysosome alkalization [14]. Additionally, this drug also reduces the levels of IL-6 in individuals with systemic lupus erythematosus and rheumatoid arthritis [15], thus hypothetically reducing the chance to develop cytokine storm and acute respiratory distress syndrome- (ARDS-) related complications [16].

Nonetheless, while small clinical trials have shown a strong effect for lopinavir/ritonavir (L/R) in reducing the viral load, along with body temperature, C-reactive protein (CRP) levels, alanine aminotransferase, and aspartate aminotransferase [17] while increasing the level of oxygen saturation and pressure, platelet, lymphocyte, leukocyte, and eosinophil counts; while reducing the extension of radiological findings [4], other studies with enhanced power have shown no reduction in viral load in relation to the L/R drug combination [18]. Additionally, other study shows that while a combination of interferon *β*-1b, L/R, and ribavirin is effective at alleviating symptoms, shortening the duration of viral shedding, and reducing hospital stay in patients with mild to moderate COVID-19, L/R alone lacks a strong regulatory ability over such disease [19].

On the other hand, the role for Chlo in COVID-19 treatment has been more controversial, as low power studies suggest that this agent is able to shorten the viral shedding stage to only 6-8 days, especially in combination with azithromycin [20], while bigger studies showed that the viral clearance rate occurred for most patients at day 10 posttreatment onset (PTO) [21] or that there were no differences in mortality or intubation risk in relation to this treatment [22]. Interestingly, none of these studies were carried out in severely ill patients, and the only study to date that was performed with this kind of patients [23] found no difference in the survival rate without transfer to the intensive care unit at day 21, overall survival, acute respiratory distress syndrome incidence, or changes in respiratory support requirements between the hydroxychloroquine and the standard care group. Finally, a study comparing L/R, Chlo, and standard care in mild to moderately ill patients found no difference in fever duration, median time from symptom onset to CT improvement, or negative conversion of PCR [24].

In such panorama, data comparing the effectiveness of both drugs and assessing its efficacy and safety in the severely ill patient is needed, as well as more studies to complement the body of knowledge about the treatment of COVID-19 with both drugs combined. In the present article, we present a retrospective study of 61 severely ill patients that received either L/R, Chlo, a combination of both, or a standard treatment without suspected antiviral agents, finding that either drug is ineffective at treating COVID-19, as they are not able to reduce mortality, hospitalization length, C-reactive protein (CRP), lactate dehydrogenase (LDH), ferritin, or d-Dimer levels or even to enhance gasometric and hematic parameters. Moreover, both drugs administered together decrease circulating lymphocytes, increase LDH and ferritin levels, and importantly, enhance mortality.

## 2. Materials and Methods

### 2.1. Patients, Treatments, and Data Collection

We retrospectively analyzed a total of 61 clinical archives belonging to patients in severe condition that were treated in the Sociedad Española de Beneficencia's Hospital, at Pachuca, Hidalgo, México, from May 1^st^ to August 30^th^. Twenty-seven patients received 2 pills of lopinavir/ritonavir (200/50 mg) twice daily (L/R group) (plus enoxaparin and dexamethasone), 11 patients received 2 pills of chloroquine (150 mg) twice daily (Chlo group) (plus enoxaparin and dexamethasone), 17 patients received a combination of the aforementioned drugs in the same doses (Chlo+L/R) (plus enoxaparin and dexamethasone), and six patients received the standard treatment (ST) with no antiviral agents, but receiving enoxaparin and dexamethasone. Treatment choice was made upon careful evaluation of each individual case to avoid adverse outcomes. The patients started their treatment as they were admitted to the hospital, so that the treatments did not occur at the same time. All the patients were confirmed positive for COVID-19 by a polymerase chain reaction (PCR), either performed in our laboratory or in other laboratories. CALL score was calculated using the online calculator that can be found in [25].

All patients signed an informed consent form prior to hospitalization, and the inclusion and exclusion criteria for our selection of the clinical records were as follows: inclusion criteria: (i) clinical records of patients who had signed a form of informed consent, (ii) clinical records of patients who had repeated measurements of the studied biomarkers, (iii) clinical records of patients that presented a severe form of the disease, (iv) age ≥ 18 years, and (v) positive PCR throat swab test for SARS-CoV-2. Exclusion criteria: (i) clinical records of patients who had any condition that made them susceptible to any of the drugs studied, (ii) clinical records of patients that were voluntarily discharged before the completion of their treatment, (iii) incomplete clinical records of patients, and (iv) negative PCR throat swab test for SARS-CoV-2.

### 2.2. Laboratory Test Biomarkers and Statistical Assessments

On the other hand, pathophysiological markers from laboratory tests were plotted and analyzed using the GraphPad Prism 5 software, and the area under the curve (AUC) was calculated for each parameter in either of the experimental-treated or standard-treated groups. The AUC parameters of the Chlo, L/R, or Chlo+L/R groups were divided into the AUC of the ST group to calculate how many times higher was the expression of each marker, except when the experimental AUCs were lower than that of the ST group, in which case they functioned as divisors. The CALL score was calculated by using the following online tool [25]. A repeated observation ANOVA or a Student's *t* test was used to assay whether a difference among groups was significant (*p* ≤ 0.05).

## 3. Results and Discussion

### 3.1. Patient's Characteristics

We analyzed 61 clinical records belonging to patients that received some kind of respiratory support and were treated with included the compassionate use (according to the European Medicines Agency recommendations) of lopinavir/ritonavir (200/50 mg) (plus enoxaparin and dexamethasone), chloroquine (150 mg) (plus enoxaparin and dexamethasone), a combination of both (plus enoxaparin and dexamethasone), and a group that received supporting therapy consisting of dexamethasone and enoxaparin but not antiviral medication, with an *n* = 27 for the first group (L/R group), *n* = 11 for the second (Chlo group), *n* = 17 for the third group (L/R+Chlo), and *n* = 6 received the latter treatment (ST group).

Numerous researchers around the globe have noticed that the elderly patients have an increased mortality rate by COVID-19, but two meta-analysis [26, 27], including more than 1,000 patients each, have shown that such age groups are at increased risk not because of their age, but from their increased prevalence of certain comorbidities like hypertension, diabetes, chronic obstructive pulmonary disease (COPD), cardiovascular disease, and cerebrovascular disease. For such reason, we first proceeded to describe the age and prevalence of comorbidities in our groups.

**Figure 1:**
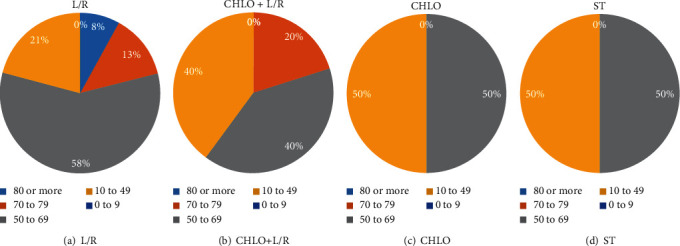
*Age groups.* Five age groups were defined and the percentage of patients belonging to each group, among the different treatment groups L/R (a), Chlo (b), L/R+Chlo (c), and ST (d), was calculated. L/R: lopinavir/ritonavir; Chlo: chloroquine; L/R+Chlo: lopinavir/ritonavir plus chloroquine; ST: standard treatment.

Patients in the L/R group were found to be generally older than those in the other groups, as such category was comprised of an 8% of patients with 80 or more years, 13% of patients with 70-79 years, 58% within the 50 to 69 years old range, and 21% from 10 to 49 years old (Figure 1(a)). Additionally, the Chlo and ST groups were younger as 50% of the patients in those groups were 50 to 69 years old, while the other half was within the 10 to 49 years old range (Figures 1(c) and 1(d)). On the other hand, the Chlo+L/R group was comprised of 40% of patients with 10 to 49 years of age, 40% with 50 to 69, and 20% from 70 to 79% (Figure 1(b)). In this way, the L/R group had the oldest patients whereas the Chlo and ST groups had the youngest.

**Figure 2:**
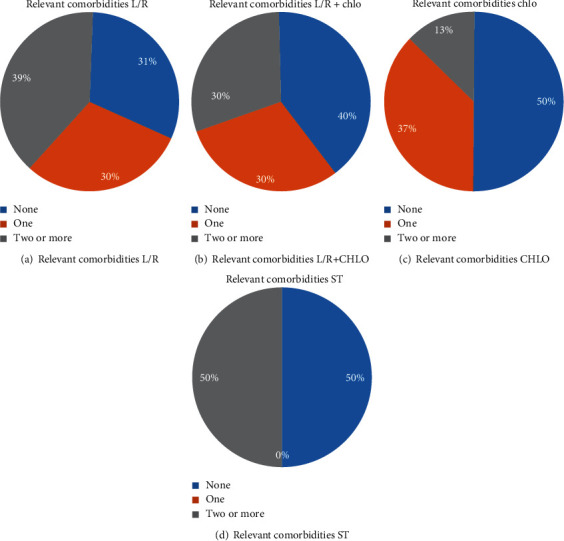
*Relevant comorbidities.* The prevalence of relevant comorbidities like hypertension, diabetes, COPD, cardiovascular disease, and chronic bronchitis was calculated for each treatment group and pooled together in three categories: none comorbidities, one comorbidity, and two or more. L/R: lopinavir/ritonavir; Chlo: chloroquine; L/R+Chlo: lopinavir/ritonavir plus chloroquine; ST: standard treatment; COPD: chronic obstructive pulmonary disease.

Moreover, the L/R group had the highest number of relevant comorbidities for COVID-19, with 39% presenting with two or more, 30% with one, and 31% with none (Figure 2(a)), which was closely followed by the L/R+Chlo group with 30% patients presenting two or more comorbidities, 30% one, and 40% none (Figure 2(b)). Half of the patients of the Chlo group had no relevant diseases, while 37% presented with one and 13% two or more (Figure 2(c)), and on the ST group, half of the patients presented no comorbidities and the other half presented two or more (Figure 2(d)).

**Figure 3:**
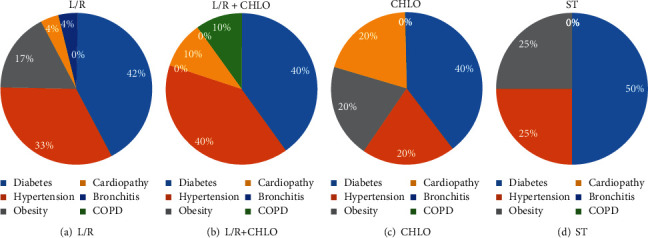
Comorbidity prevalence per group. The prevalence of relevant comorbidities like hypertension, diabetes, COPD, cardiovascular disease, and chronic bronchitis was calculated for each treatment group. L/R: lopinavir/ritonavir; Chlo: chloroquine; L/R+Chlo: lopinavir/ritonavir plus chloroquine; ST: standard treatment; COPD: chronic obstructive pulmonary disease.

Diabetes was the most prevalent comorbidity within our patients among groups, ranging from 40% to 50% (Figure 3); hypertension followed in prevalence, ranging from 20 to 40% and being more represented in the L/R+Chlo group (Figure 3(b)), and obesity was more highly represented in the ST group (Figure 3(d)). As hypertension [28], type 2 diabetes [29], and obesity [30] have been vastly prevalent in México, these comorbidities were expected to be regularly distributed among our four groups.

### 3.2. Survival and Hospital Stay

**Figure 4:**
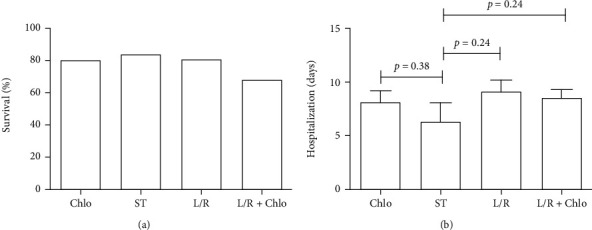
Survival and hospital stay length. The overall survival rate was estimated disregarding the length of hospital stay (a), and the hospital stay was calculated only for those patients that survived (b). A Student's *t* test was performed, considering significant a *p* ≤ 0.05. L/R: lopinavir/ritonavir; Chlo: chloroquine; L/R+Chlo: lopinavir/ritonavir plus chloroquine; ST: standard treatment.

Million and colleagues [21] reported an enhanced survival rate for patients treated with Chlo, while other researchers found that this drug was able to shorten the length of the viral reproduction stage of the disease [6]. On the other hand, L/R also seems to reduce mortality [18], but its benefit in reducing hospital stay is not clear [19]. Consequently, our data shows an equal survival in the Chlo (80%), ST (83%), and L/R (80.7%). Strikingly, the L/R+Chlo group showed the lowest survival with just 68% (Figure 4(a)). Such results stand out as L/R+Chlo-treated patients were not the most advanced in age (Figure 1(b)), nor the ones with more comorbidities (Figure 2(b)). Moreover, being that the patients in the L/R group were the eldest and possessed more relevant comorbidities (Figures 1(a) and 2(a)), we think that the role for L/R in modulating survival should not be ruled out. In regard to the length of the hospitalization, there was no difference between any of the groups (Figure 4(b)), with Chlo, ST, L/R, and L/R+Chlo groups averaging 8.1, 6.3, 9.0, and 8.4 days, respectively.

### 3.3. Modulation of the Pathology Biomarkers

As COVID-19 is a disease that in its severe forms produces lung and systemic inflammation, systemic and lung tissue damage, and coagulopathy, several markers like CRP, LDH, d-Dimer, and ferritin have been proposed as predictors of disease severity and mortality. Furthermore, LDH, lymphocytes, age, and comorbidities have been used to calculate the risk of developing severe COVID-19 [31], in a new clinical score termed CALL score, which stands for comorbidities, age, LDH, and lymphocytes [25].

**Figure 5:**
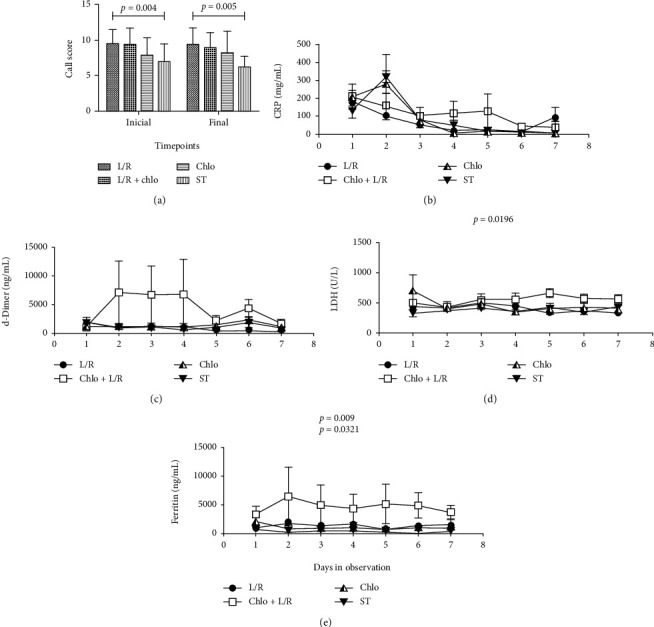
Modulation of pathology biomarkers. The CALL score was calculated for each patient in each group (a), and the concentration of C-reactive protein (CRP) (b), d-Dimer (c), lactate dehydrogenase (LDH) (d), and ferritin (e) was plotted into a histogram for each patient from day 1 to day 7. A Student's *t* tests (a) and ANOVA tests (b–e) were performed comparing each treatment against ST, a *p* ≤ 0.05 was considered significant. L/R: lopinavir/ritonavir; Chlo: chloroquine; L/R+Chlo: lopinavir/ritonavir plus chloroquine; ST: standard treatment.

In such an understanding, we plotted the initial (hospitalization onset) and final (end of hospitalization) CALL score values of each patient belonging to any of our groups. A Student's *t* test analysis confirmed that there was no significant difference between the initial and final time points for any of the treatments. Among groups, only the L/R-treated patients were shown to be in a worse condition than the ST patients at the initial time point, and this difference was still present when comparing both treatments at the end points (Figure 5(a)).

Furthermore, blood levels of the aforementioned markers were plotted into a histogram to calculate the area under the curve (AUC) and compare its value for each treatment with that of the ST group by means of a repeated observations ANOVA test. The Chlo+L/R treatment was unable to produce a significant modulation of CRP (1.22-fold higher) (Figure 5(b), Table 1), or d-Dimer (Figure 5(c), Table 2) (4.1-fold higher), but produced a significant increase in LDH levels (1.42-fold higher) (Figure 5(d), Table 3), and especially in ferritin concentrations (13.58-fold) (Figure 5(e), Table 4). In comparison, Chlo-treated patients did not present any significant change in the aforementioned parameters (Figure 5), while the L/R group presented a significant increase of ferritin (Figure 5(e), Table 4).

**Figure 6:**
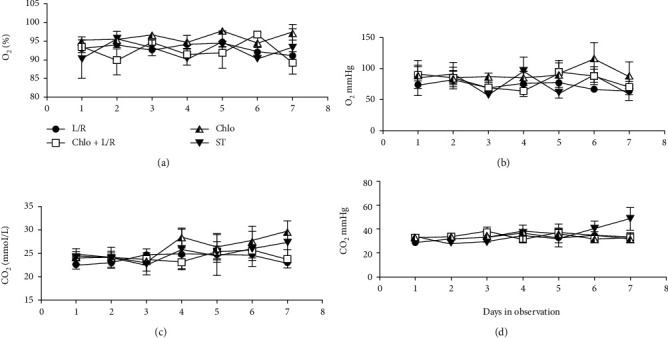
Respiratory function. Oxygen saturation (a) and pressure (b) as well as total CO_2_ (c) and CO_2_ pressure (d) measurements were plotted into a histogram for each patient from day 1 to day 7. ANOVA tests were performed comparing each treatment against ST, a *p* ≤ 0.05 was considered significant. L/R: lopinavir/ritonavir; Chlo: chloroquine; L/R+Chlo: lopinavir/ritonavir plus chloroquine; ST: standard treatment.

**Figure 7:**
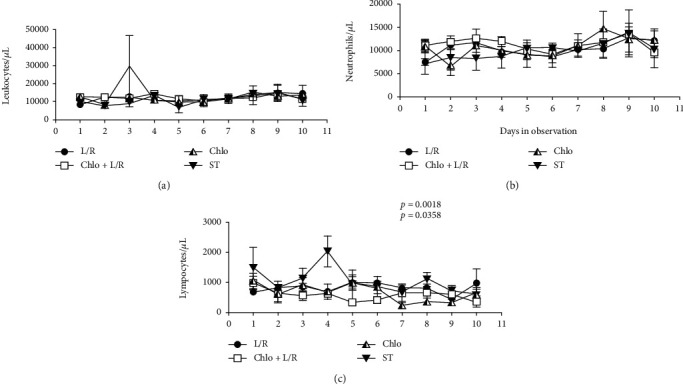
White blood cells. Total leukocytes (a), neutrophils (b), and lymphocytes (c) were measured and plotted into a histogram for each patient from day 1 to day 7. ANOVA tests were performed comparing each treatment against ST, a *p* ≤ 0.05 was considered significant. L/R: lopinavir/ritonavir; Chlo: chloroquine; L/R+Chlo: lopinavir/ritonavir plus chloroquine; ST: standard treatment; AUC: area under the curve.

No significant difference was observed between groups regarding oxygen saturation (Figure 6(a), Table 5), pressure (Figure 6(b), Table 6), total CO_2_ (Figure 6(c), Table 7), CO_2_ pressure (Figure 6(d), Table 8), total leukocytes (Figure 7(a), Table 9), or neutrophils (Figure 7(b), Table 10). However, both the Chlo and Chlo+L/R groups had significantly smaller populations of lymphocytes (Figure 7(c), Table 11).

Taken together, our results show a low efficacy for both chloroquine and L/R, alone or together, in the treatment of COVID-19, as they produced no significant differences with respect to the standard treatment.

## 4. Limitations of the Study

Despite the interesting population that was studied, the present research is limited by its retrospective nature, as this fact does not allow for a proper randomization of the treatments, or the use of a placebo control. Moreover, the study may have been benefited from an increased power, as this study covers a segment of population (severely ill patients) that has been neglected by current research on the topic. Finally, increased follow-up times may also enhance our results, as some studies are evaluating fever, relief of respiratory symptoms, respiration rate, and fingertip blood oxygenation at day 28 post symptom onset as a means to study complete recovery of the patients [32]. The aforementioned limitations could be easily overcome by performing the study in a prospective manner and by collaborating with other health centers to increase the power of the study.

## 5. Discussion

Nine months after the appearance of the SARS-CoV-2 pandemics, much data is needed regarding effective pharmacotherapies [33]. Although some medical and health care associations have recommended the use of Chlo, hydroxychloroquine, or L/R in severely ill older patients [34], other more conservative agencies recommend against the use of such drugs [35] in the treatment of COVID-19. The main rationale for this recommendation is the lack of proofs regarding such treatments' effectiveness and safety, especially in hospitalized patients. Large international clinical trials like “Solidarity,” from the World Health Organization (WHO) have even suspended the parts of their studies regarding chloroquine and lopinavir/ritonavir, due to repeated ineffectiveness [36].

In the present study, we did not detect any difference in hospitalization length, CRP, or d-dimer production, oxygen saturation and pressure, total CO_2_ or CO_2_ pressure, or even neutrophils and total leukocytes between the Chlo, L/R, or Chlo+L/R-treated patients in comparison to those receiving the standard treatment. On the other hand, we found that the treatment with Chlo+L/R enhanced the levels of LDH and ferritin, while reducing total lymphocytes and survival and that L/R alone increases ferritin. For such reasons, we think that both L/R and Chlo may not be effective to treat severely ill COVID-19 patients and that the combination of both drugs may worsen the pathology.

Although the anti-COVID-19 efficacy of Chlo in mild to moderately ill patients has been a matter of intense debate, it has been increasingly clear that this drug does not produce a significant modulation of the severe form of the disease [37]. Rather, this drug may increase the risk of patients to develop a prolonged repolarization phase within the QT interval of the heart beating, thus causing Torsades de Pointes, which is a kind of ventricular arrhythmia [38]. Moreover, this drug has been reported to worsen symptoms of Chikungunya [39] and influenza [40], despite the positive results that this drug offers when assayed *in vitro* for these and other viruses [38]. For such reasons, it is important to develop a stronger body of evidence regarding the repurpose of such drug in the treatment of any new viral disease, like COVID-19, especially in vulnerable sectors of the population [41].

On the other hand, L/R is a peptidomimetic molecule that inhibits the activity of viral 3-chymotrypsin-like protease (3CL^pro^) by occupying its active site, causing a competitive inhibition [8]. Such enzyme is important for HIV replication, and as SARS-CoV-2 expresses it [9], it has been thought that this drug may be able to inhibit such virus' replication. However, to our knowledge, no *in vitro* experiments have been done to confirm this rationale. Even when L/R has shown an enhanced ability to treat SARS and MERS, this drugs were assayed for effectivity after demonstration an *in vitro* anti-SARS [42] and MERS [43] activity. The efficacy of this drug is a matter of intense debate, with small trials finding reductions, pathophysiological markers, and viral loads [17], or even in radiological findings [4], and bigger trials finding no difference in most or any parameters measured [18, 44]. Moreover, such trials have shown a significant increase in gastrointestinal adverse effects in relation to L/R [18, 44], in such a way that the safety concerns about this drug in COVID-19-treatments may outweigh their potential benefits [45].

Finally, both L/R and Chlo have been considered as potential inductors of prolonged QT interval and Torsade de Pointes, and their potential for this adverse effect is thought to be enhanced when these drugs are administered together [46], and an interesting study by the French Network of Pharmacovigilance Centers [47] demonstrated an increased incidence of cardiac adverse events in one month in relation to the treatment with these drugs.

## 6. Conclusions

In this retrospective study, we showed that both L/R and Chlo may not be effective in the modulation of severe COVID-19 and that together may worsen the overall pathology. Considering all the information about these drugs' potential adverse effects together with our observations, we think that the effects of Chlo and L/R on COVID-19 should be studied not only with the aim of finding an effective treatment, but with the intention to thoroughly describe their safety profile.

## Figures and Tables

**Table 1:** C-reactive protein levels.

CRP	AUC	Fold increase/decrease	*p* value
Chlo+L/R	674	1.22-fold higher	*p* = 0.5227
L/R	337.8	1.63-fold lower	*p* = 0.3825
Chlo	264.6	2.08-fold lower	*p* = 0.9397
ST	552.2		

The area under the curve (AUC) was calculated and compared to that of the ST group. ANOVA tests were performed, considering significant a *p* ≤ 0.05. L/R: lopinavir/ritonavir; Chlo: chloroquine; L/R+Chlo: lopinavir/ritonavir plus chloroquine; ST: standard treatment; AUC: area under the curve; CRP: C-reactive protein; LDH: lactate dehydrogenase.

**Table 2:** d-Dimer levels.

d-Dimer	AUC	Fold increase/decrease	*p* value
Chlo+L/R	28659	4.10-fold higher	*p* = 0.5438
L/R	5523	1.26-fold lower	*p* = 0.0648
Chlo	8442	1.21-fold higher	*p* = 0.6188
ST	6975		

The area under the curve (AUC) was calculated and compared to that of the ST group. ANOVA tests were performed, considering significant a *p* ≤ 0.05. L/R: lopinavir/ritonavir; Chlo: chloroquine; L/R+Chlo: lopinavir/ritonavir plus chloroquine; ST: standard treatment; AUC: area under the curve; CRP: C-reactive protein; LDH: lactate dehydrogenase.

**Table 3:** Lactate dehydrogenase levels.

LDH	AUC	Fold increase/decrease	*p* value
Chlo+L/R	3307	1.42-fold higher	*p* = 0.0196
L/R	2420	1.04-fold higher	*p* = 0.7667
Chlo	2654	1.14-fold higher	*p* = 0.6264
ST	2317		

The area under the curve (AUC) was calculated and compared to that of the ST group. ANOVA tests were performed, considering significant a *p* ≤ 0.05. L/R: lopinavir/ritonavir; Chlo: chloroquine; L/R+Chlo: lopinavir/ritonavir plus chloroquine; ST: standard treatment; AUC: area under the curve; CRP: C-reactive protein; LDH: lactate dehydrogenase.

**Table 4:** Ferritin levels.

Ferritin	AUC	Fold increase/decrease	*p* value
Chlo+L/R	29664	13.58-fold higher	*p* = 0.0321
L/R	8431	3.86-fold higher	*p* = 0.009
Chlo	6304	2.88-fold higher	*p* = 0.1272
ST	2184		

The area under the curve (AUC) was calculated and compared to that of the ST group. ANOVA tests were performed, considering significant a *p* ≤ 0.05. L/R: lopinavir/ritonavir; Chlo: chloroquine; L/R+Chlo: lopinavir/ritonavir plus chloroquine; ST: standard treatment; AUC: area under the curve; CRP: C-reactive protein; LDH: lactate dehydrogenase.

**Table 5:** Oxygen saturation.

Oxygen saturation	AUC	Fold increase/decrease	*p* value
Chlo+L/R	555.8	1.00-fold lower	*p* = 0.5512
L/R	559.4	1.00-fold higher	*p* = 0.6610
Chlo	575.2	1.03-fold higher	*p* = 0.1060
ST	556.3		

The area under the curve (AUC) was calculated and compared to that of the ST group. ANOVA tests were performed, considering significant a *p* ≤ 0.05. L/R: lopinavir/ritonavir; Chlo: chloroquine; L/R+Chlo: lopinavir/ritonavir plus chloroquine; ST: standard treatment; AUC: area under the curve; CRP: C-reactive protein; LDH: lactate dehydrogenase.

**Table 6:** Oxygen pressure.

Oxygen pressure	AUC	Fold increase/decrease	*p* value
Chlo+L/R	481.2	1.03-fold higher	*p* = 0.9395
L/R	440.6	1.05-fold lower	*p* = 0.6647
Chlo	552.1	1.19-fold higher	*p* = 0.0336
ST	463.3		

The area under the curve (AUC) was calculated and compared to that of the ST group. ANOVA tests were performed, considering significant a *p* ≤ 0.05. L/R: lopinavir/ritonavir; Chlo: chloroquine; L/R+Chlo: lopinavir/ritonavir plus chloroquine; ST: standard treatment; AUC: area under the curve; CRP: C-reactive protein; LDH: lactate dehydrogenase.

**Table 7:** Total carbon dioxide.

Total CO_2_	AUC	Fold increase/decrease	*p* value
Chlo+L/R	145.9	1.02-fold lower	*p* = 0.3636
L/R	145	1.02-fold lower	*p* = 0.3620
Chlo	157.2	1.05-fold higher	*p* = 0.5638
ST	148.9		

The area under the curve (AUC) was calculated and compared to that of the ST group. ANOVA tests were performed, considering significant a *p* ≤ 0.05. L/R: lopinavir/ritonavir; Chlo: chloroquine; L/R+Chlo: lopinavir/ritonavir plus chloroquine; ST: standard treatment; AUC: area under the curve; CRP: C-reactive protein; LDH: lactate dehydrogenase.

**Table 8:** Carbon dioxide pressure.

CO_2_ pressure	AUC	Fold increase/decrease	*p* value
Chlo+L/R	203.2	1.00	*p* = 0.3019
L/R	200.4	1.02-fold lower	*p* = 0.5647
Chlo	206.3	1.00-fold higher	*p* = 0.7635
ST	205.2		

The area under the curve (AUC) was calculated and compared to that of the ST group. ANOVA tests were performed, considering significant a *p* ≤ 0.05. L/R: lopinavir/ritonavir; Chlo: chloroquine; L/R+Chlo: lopinavir/ritonavir plus chloroquine; ST: standard treatment; AUC: area under the curve; CRP: C-reactive protein; LDH: lactate dehydrogenase.

**Table 9:** 

Total leukocytes	AUC	Fold increase/decrease	*p* value
Chlo+L/R	110330	1.11-fold higher	*p* = 0.2066
L/R	107941	1.08-fold higher	*p* = 0.3558
Chlo	122758	1.23-fold higher	*p* = 0.3883
ST	99217		

The area under the curve (AUC) was calculated and compared to that of the ST group. ANOVA tests were performed, considering significant a *p* ≤ 0.05. L/R: lopinavir/ritonavir; Chlo: chloroquine; L/R+Chlo: lopinavir/ritonavir plus chloroquine; ST: standard treatment; AUC: area under the curve; CRP: C-reactive protein; LDH: lactate dehydrogenase.

**Table 10:** 

Neutrophils	AUC	Fold increase/decrease	*p* value
Chlo+L/R	102270	1.13-fold higher	*p* = 0.1533
L/R	93912	1.04-fold higher	*p* = 0.6386
Chlo	95946	1.06-fold higher	*p* = 0.4507
ST	90233		

The area under the curve (AUC) was calculated and compared to that of the ST group. ANOVA tests were performed, considering significant a *p* ≤ 0.05. L/R: lopinavir/ritonavir; Chlo: chloroquine; L/R+Chlo: lopinavir/ritonavir plus chloroquine; ST: standard treatment; AUC: area under the curve; CRP: C-reactive protein; LDH: lactate dehydrogenase.

**Table 11:** 

Lymphocytes	AUC	Fold increase/decrease	*p* value
Chlo+L/R	5128	1.82-fold lower	*p* = 0.0018
L/R	7319	1.28-fold lower	*p* = 0.0604
Chlo	5886	1.59-fold lower	*p* = 0.0358
ST	9375		

The area under the curve (AUC) was calculated and compared to that of the ST group. ANOVA tests were performed, considering significant a *p* ≤ 0.05. L/R: lopinavir/ritonavir; Chlo: chloroquine; L/R+Chlo: lopinavir/ritonavir plus chloroquine; ST: standard treatment; AUC: area under the curve; CRP: C-reactive protein; LDH: lactate dehydrogenase.

## Data Availability

As data was directly extracted from the clinical files of our hospital, where not only scientific but also personal information about our patients can be found, we cannot publish these data.
